# Mechanisms of drug resistance to immune checkpoint inhibitors in non-small cell lung cancer

**DOI:** 10.3389/fimmu.2023.1127071

**Published:** 2023-02-08

**Authors:** Kexun Zhou, Shuo Li, Yi Zhao, Ke Cheng

**Affiliations:** ^1^ Abdominal Oncology Ward, Division of Medical Oncology, Cancer Center, State Key Laboratory of Biological Therapy, West China Hospital, Sichuan University, Chengdu, China; ^2^ Abdominal Oncology Ward, Division of Radiation Oncology, Cancer Center, State Key Laboratory of Biological Therapy, West China Hospital, Sichuan University, Chengdu, China; ^3^ Department of Thoracic Surgery, West China Hospital, Sichuan University, Chengdu, China; ^4^ Lung Cancer Center, West China Hospital Sichuan University, Chengdu, China; ^5^ The First Clinical Medical College of Lanzhou University, Lanzhou University, Lanzhou, China

**Keywords:** immunotherapy, resistance, mechanism, tumor microenvironment, lung cancer

## Abstract

Immune checkpoint inhibitors (ICIs) in the form of anti-CTLA-4 and anti-PD-1/PD-L1 have become the frontier of cancer treatment and successfully prolonged the survival of patients with advanced non-small cell lung cancer (NSCLC). But the efficacy varies among different patient population, and many patients succumb to disease progression after an initial response to ICIs. Current research highlights the heterogeneity of resistance mechanisms and the critical role of tumor microenvironment (TME) in ICIs resistance. In this review, we discussed the mechanisms of ICIs resistance in NSCLC, and proposed strategies to overcome resistance.

## 1 Introduction

Lung cancer is currently the second most frequently diagnosed cancer and the leading cause of cancer mortality globally (1). Immune checkpoint inhibitors (ICIs) have changed the cancer treatment paradigm and become the standard care for many cancers, including lung cancer. Whether as monotherapy or in combination, ICIs have shown inspiring efficacy in advanced non-oncogene-driven non-small cell lung cancer (NSCLC), extensive-stage small cell cancer, as well as unresectable stage III NSCLC (2, 3). In addition, the role of ICIs in neoadjuvant chemotherapy for resectable lung cancer is gradually being explored (4–7). To date, ICIs targeting three different immune checkpoints have been approved by the US Food and Drug Administration (FDA) for cancer therapy, namely cytotoxic T-lymphocyte-associated protein 4 (CTLA-4) antibody, programmed cell death protein 1 (PD-1) antibody, and programmed death ligand 1 (PD-L1) antibody. Over years, the application of anti-PD-1/PD-L1 antibodies has greatly surpassed anti-CTLA-4, due to superior efficacy and safety profiles (8).

Although ICIs have significantly improved the survival in patients, the efficacy varied in different patient population, as a large proportion of patients had poor response to ICIs and succumb to disease progression, especially with ICIs monotherapy. The disparities represented a critical knowledge gap and attracted widespread attention, driving efforts to address this issue. Therefore, it is important to review the current mechanism of resistance and reveal how to ‘activate’ non-responders. In this review, we summarized the mechanisms of ICIs resistance in lung cancer and the factors which were associated with the clinical effects, aiming to promote better use of ICIs in lung cancer treatment.

## 2 Immunotherapy overview

### 2.1 Anti-tumor immunity

Initiation of the anti-tumor immune response occurs when cells of the innate immune system become alerted to the presence of tumor, the signal of which may be conveyed by pro-inflammatory molecules, chemokines of the tumor site, and cells of the innate immune system (9). Tumor cells are recognized by the intrinsic immune cells, and the recognition mechanism is inconclusive (10). Once recognized, the intrinsic immune cells (e.g., natural killer (NK) T cells, γδ T cells, and macrophages) will trigger initial intrinsic immunity and interferon (IFN) secretion, which promotes chemokines production, and the recruitment of immune cells. IFN could activate a series of IFN-dependent processes (e.g., anti-proliferative, pro-apoptotic, anti-angiogenic) to kill tumor cells (9). The dead tumor cells during this process makes tumor antigens available, driving specific immunity.

**Figure 1:**
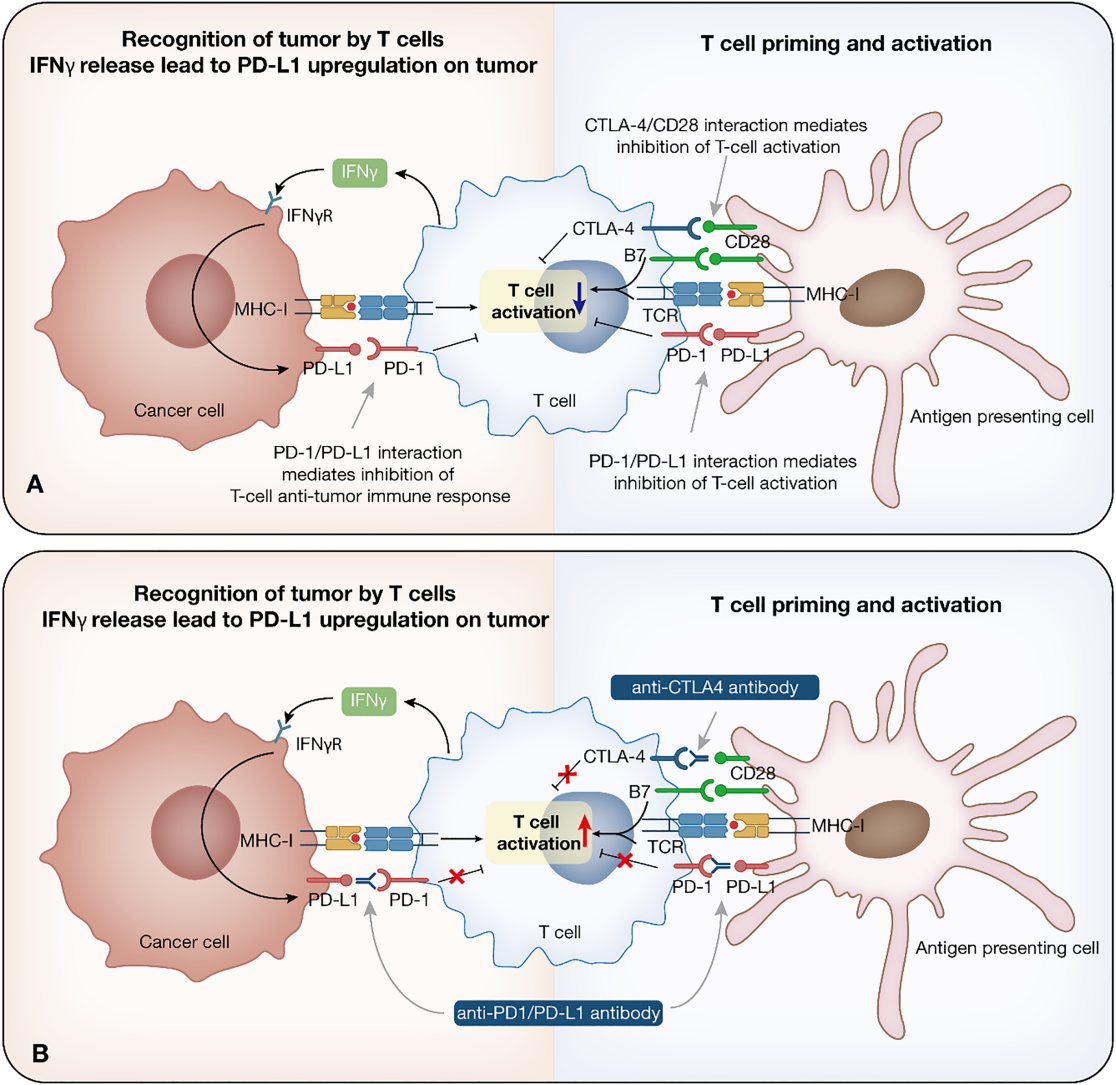
Immune checkpoint inhibitor therapy. Immune response to cancer and relationship among cancer cells, T cells and antigen presenting cells. **(A)** Tumor cells are recognized by the intrinsic immune cells. Once recognized, immune cells trigger initial intrinsic immunity and IFN secretion, promoting chemokines production, and the recruitment of immune cells. Mature DCs take up tumor antigens, which finally presented as MHC-I antigenic tumor peptides complex, migrate to the draining lymph nodes, where initial tumor-specific Th1 CD4^+^ T cells and CTLs are induced by antigen cross-presentation. IL-2 and IL-15 help to maintain CTLs production and viability. **(B)** Mechanism of anti-PD-1/PD-L1/CTLA-4 antibody. PD-1 is expressed on T cells, while PD-L1 is expressed on tumor cells. These two form the PD-1/PD-L1 axis that mediates T cell function inhibition. The function of anti-PD-1/PD-L1 antibodies lies in restoring the T cell effect by blocking the PD-1/PD-L1 interaction. CTLA-4 on T cells binds to B7 ligand on the APC, leading to immunosuppression. Anti-CTLA-4 antibodies inhibit the binding between CTLA-4 and B7, which prolongs T cell activation, restore T cell proliferation, and establish an immune response to tumor-associated antigens. IFN, interferon; DC, dendritic cell; MHC-I, major histocompatibility complex class I; CTL, cytotoxic T lymphocyte; APC, antigen-presenting cell.

Immature dendritic cells (DCs) are recruited to the tumor and triggered by cytokine exposure or intercellular interaction. Mature DCs take up tumor antigens, which finally presented as major histocompatibility complex class I (MHC-I) antigenic tumor peptides complex on DCs surface, migrate to the draining lymph nodes, where initial tumor-specific T helper type 1 (Th1) CD4^+^ T cells and CD8^+^ cytotoxic T lymphocytes (CTLs) are induced by antigen cross-presentation (9). Other antigen-presenting cells (APCs) are also involved in the process. CD4^+^ and CD8^+^ T cells migrate to the tumor site. CD4^+^ T cells produce IL-2, which, together with IL-15 produced by the host cells, helps to maintain CTLs production and viability. T cells can also release large amounts of IFN, which, together with intrinsic immune cells, kill tumors and induce tumor regression through numerous mechanisms (9, 11) (**Figure 1A**).

Tumor regression announces the victory of the immune system in the fight against tumors, but this is not the end. The ‘3Es’ hypothesis describes the continuous dynamic changes of immune cells and tumor cells in the three sequential processes of elimination, equilibrium, and escape (9, 12). Some tumor variants can escape from killing and enter dynamic equilibrium, a phase in which killing factors within the tumor exert a solid and continuous selective pressure on tumor cells. Due to genomic instability, although some of the escaped variants are destroyed, new variants carrying different mutations re-emerge and enhance resistance to immune attack. The result of the equilibrium is the generation of a new population of tumor clones with reduced immunogenicity and nearly ineffective anti-cancer defense from heterogeneous parents. It is well established that anti-cancer defenses are ineffective in solid tumors (13).

### 2.2 Immune checkpoint inhibitors

To survive and proliferate, tumors employ various strategies to escape anti-tumor immunity. Bypassing, overriding, or abolishing is the key mechanisms to restart tumor elimination, and restore T cell-mediated anti-tumor immunity in current cancer immunotherapies (14). T cells exerting immune effects requires multiple sequential steps, including successful antigen presentation and recognition, activation and proliferation, transport, and execution of killing effects. Each step is regulated by the balance between stimulatory and inhibitory signals.

Immune checkpoints are a range of inhibitory molecules that physiologically balance co-stimulatory pathways to fine-tune immune effects and prevent further immune responses. They involve inhibitory receptors and their ligands. Under normal physiological conditions, immune checkpoints are essential for maintaining self-tolerance, regulating the duration and intensity of normal physiological immune responses, and minimizing collateral damage to healthy tissues (8). Tumors can also express immune checkpoints to escape from the elimination phase and promote progression. Meanwhile, it also provides a chance to restore the anti-tumor immunity of T cells through antibodies that bind to immune checkpoints, and block inhibitory receptor-ligand interactions. These mechanisms established a cornerstone for the development of ICIs.

Currently, ICIs that have been approved by the FDA target three different immune checkpoints, PD-1, PD-L1, and CTLA-4. The bind between CD28 on T cells and B7 ligand on MHC cells is essential for T cell function, further induction, and amplification of the immune response (15). In response to T cell activation, CTLA-4 is induced to upregulate on T cells, competing with higher affinity for binding to B7 ligand on the APC, which leads to downregulation of T cells and immunosuppression (16). Anti-CTLA-4 antibodies inhibit the binding between CTLA-4 and B7, which could prolong T cell activation, restore T cell proliferation, and establish an immune response to tumor-associated antigens (TAAs) (17). Ipilimumab is the currently approved ICI targeting CTLA-4.

PD-1/PD-L1 inhibitors mainly block the feedback mechanism between T cells and tumor cells in TME. PD-1 is expressed on T cells, while PD-L1 is expressed on tumor cells. These two form the PD-1/PD-L1 axis that mediates T cell function inhibition. The function of anti-PD-1/PD-L1 antibodies lies in restoring the T cell effect by blocking the PD-1/PD-L1 interaction (18). Currently, Nivolumab and Pembrolizumab target PD-1, while Atezolizumab, Durvalumab, and Avelumab target PD-L1 (2) (**Figure 1B**).

## 3 Drug resistance mechanism

To date, clinically approved ICIs targeting PD-1/PD-L1 and CTLA-4 have shown promising efficacy and became the standard treatments for various cancers, such as advanced melanoma, NSCLC, and solid tumors with microsatellite instability (2, 12, 19). However, substantial evidence indicated that responses to ICIs varied widely among different patient population. For patients with NSCLC, the efficacy of ICIs was largely associated with the expression of PD-L1, as well as tumor percentage score (TPS). For example, the results of CheckMate-227 study suggested, treated with nivolumab in combination with ipilimumab, a response rate (ORR) of 35.9% was achieved in NSCLC patients with PD-L1 TPS ≥1% (20). These findings were born out in KEYNOTE-598 study, as pembrolizumab combined with ipilimumab provided an ORR of up to 45.4% in patients with PD-L1 TPS ≥50%. Some candidates did not benefit from the ICIs, and even suffered progression during the administration of ICIs (21).

**Figure 2:**
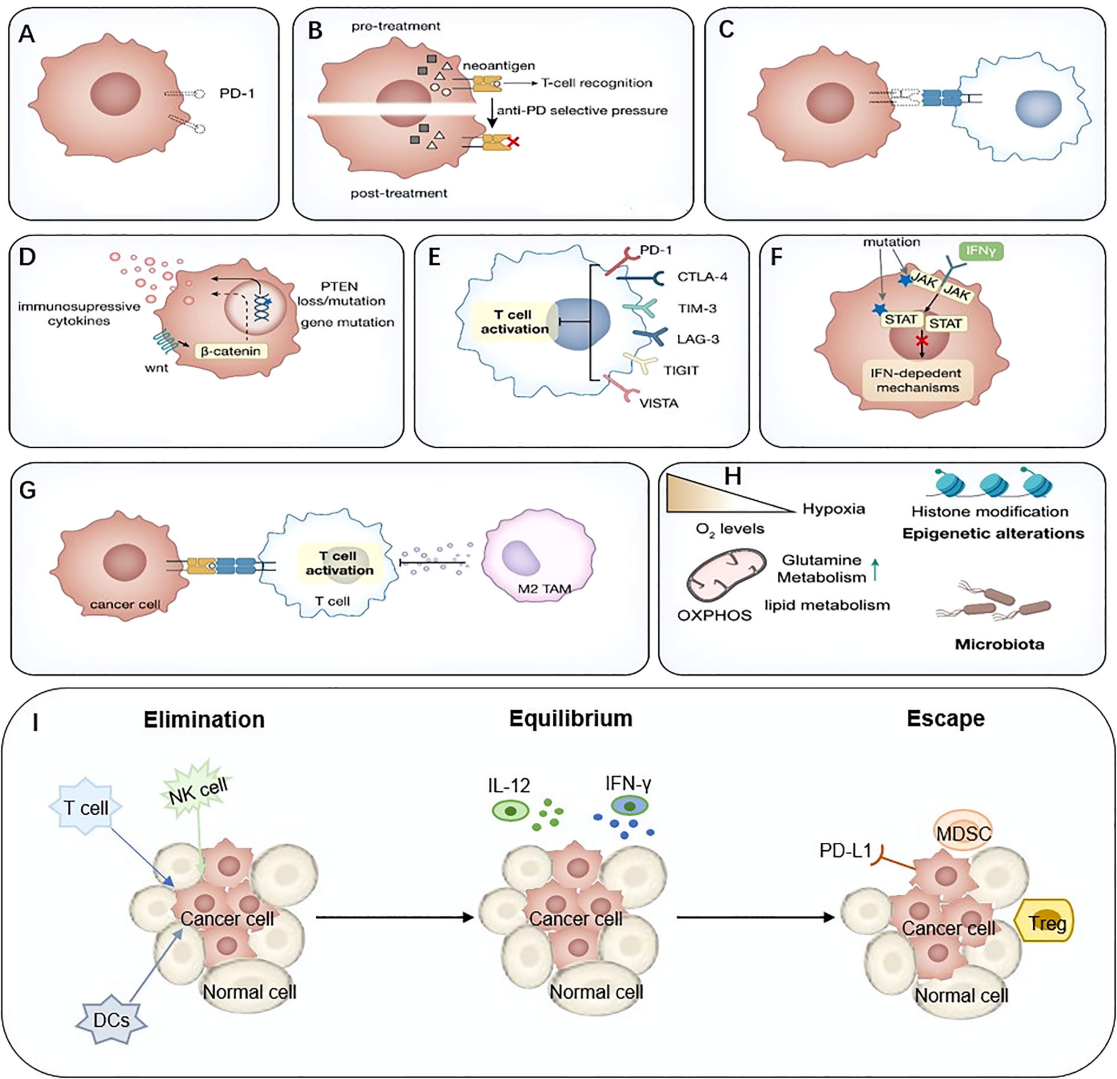
Mechanisms of immune-mediated resistance to ICIs. **(A)** Lack of PD-L1, **(B)** Neoantigen-depletion, **(C)** Antigen presentation defect, **(D)** Tumor-mediated immune-suppression or –exclusion, **(E)** Immune inhibitory receptors, **(F)** Abnormal IFN signaling pathway, **(G)** Immunosuppressive cells, **(H)** Metabolic reprogramming, **(I)** The ‘3Es’ hypothesis. IFN, interferon; ICIs, immune checkpoint inhibitor.

Extensive studies have been conducted to explore the mechanisms of ICIs resistance. From the perspective of the anti-tumor immunity process, ICIs resistance can arise at all stages, from antigen uptake and presentation to T cell killing. For example, antigen loss, impaired antigen presentation, reduced T cell infiltration, lack of PD-L1 expression, and other immune co-suppressive molecules were associated with response to ICIs. In addition, intrinsic immunity components, especially IFN, which plays a crucial role in anti-tumor immunity, are related to ICIs resistance. Other components in tumor microenvironment (TME), such as immunosuppressive molecules, immunosuppressive cells, and neutralizing antibodies for PD-1 antibodies, can also influence ICIs response. In addition, the intestinal microbiota is involved in such progress. However, due to the lack of sufficient data the mechanisms remain unclear at present (22, 23) (**Figure 2**).

### 3.1 PD-L1 expression deficiency

The main effect of PD-1/PD-L1 inhibitors is to block the PD-1/PD-L1 axis. Based on the expression pattern of tumor-infiltrating T cells (TIL) and B7-H1 (PD-L1), the tumor immune microenvironment was classified as four types: T1 (B7-H1^−^, TIL^−^), T2 (B7-H1^+^, TIL^+^), T3 (B7-H1^−^, TIL^+^), and T4 (B7-H1^+^, TIL^−^) (24, 25), among of which, the Type II was most likely to respond to ICIs. This hypothesis is supported by clinical practice, as patients with PD-L1 positive were associated with better response to anti-PD-1 therapy. Despite of this, but the efficacy of ICIs limited in a subset of these patients, suggesting the existence of other immunosuppressive pathways. For the other three types, it can be inferred that the T1 type should be the least effective, as it is lack of both TIL and B7-H1. And for T3 type, it is identified as infiltrated with TIL but lack of PD-L1 expression, which possibly due to the lack of IFN-γ production by effector T cells ((Teffs), a sign of cellular dysfunction (24). Unfortunately, the proportion of patients with T2 is not high. Data from previous studies suggest that only 17% of NSCLC patients have type II tumor immune microenvironment. Furthermore, more than half of tumors lack PD-L1 expression or TIL infiltration (25–27). Interestingly, some studies suggest that neither knockout nor overexpression of PD-L1 in tumor cells affected the efficacy of PD-L1 inhibitors, but it is the PD-L1 located on immune cells (DCs, macrophages) that correlates with the efficacy of anti-PD-1 alone or in combination with anti-CTLA-4. Although this finding remains to be further validated, it suggests a role of other cellular components within TME in the efficacy of ICIs (28).

Assessment of tissue PD-L1 expression has predictive utility for treatment outcome (29). Notably, current clinical trials largely examined the predictive value of PD-L1 expression on tumor cells. This remains some limitations. For example, due to the dynamic progression of tumors, this method can only specify tumors’ PD-L1 profile at a particular time. In addition, due to the limitation of biopsy sample size and tumor spatial, as well as temporal heterogeneity, biopsies may miss tumor tissues with high PD-L1 expression. This may explain the response of a proportion of PD-L1-negative patients to ICIs (30, 31). Existing evidence suggested, besides of tumor cells, the PD-L1 expression on tumor-infiltrating immune cells (e.g., macrophages, DCs, neutrophils, and T cells) or elsewhere could also be assessed. By using immunohistochemistry (IHC), Taube JM et al. found the expression of PD-L1 was also observed in the TILs and associated macrophages/histiocytes, which was associated with the outcome of patients with metastatic melanoma (32). Meanwhile, it has been established that PD-L1-expressing tumor-associated macrophages (TAMs) could impair the function of NK cells, and contribute to the inefficient sensitivity to PD-1 inhibitors (33). Furthermore, study performed by Hinterleitner C and their co-workers have found the interaction between the blood platelets and lung cancer cells. Specially, PD-L1 expression on platelets could not only reflect that on tumor, but also inhibit CD4^+^ and CD8^+^ T cells, which provided a new perspective to overcome the limitations of traditional quantification of PD-L1 expression on tumor cells (34). Further understanding the host immune system and TME will better identify patients who will benefit from these agents. For patients with high PD-L1 expression but insensitive to ICIs, other resistance mechanisms may play a leading role. For example, EGFR and HER-2 mutations, and the fusion of ALK, ROS1, RET, MET could define NSCLC subsets with minimal benefit from ICIs, despite of high PD-L1 expression (35).

On the other hand, in addition to membrane-bound form, PD-L1 could be secreted as a truncated form, which called soluble PD-L1. Preclinical evidence suggested that this form also contributed to the resistance to immunotherapy (36, 37). In a retrospective study, blood samples from patients treated with ICIs were analyzed. The results revealed that soluble PD-L1 levels could be regarded as an independent prognostic factor, as inferior outcomes were observed in patients with high soluble PD-L1 (38). This finding has been conquered in other studies (39–41). But the potential mechanism needs to be further investigated.

### 3.2 Antigen presentation disorders and antigen deficiency

Tumor cells may have defects in the processing and presentation of antigen and antigen, resulting in the inability of the immune system to detect new antigens and initiate an effective immune response. Each human leukocyte antigen class I (HLA-I) molecule binds specific peptides derived from intracellular proteins and is expressed on the cell surface for presentation to CD8^+^ T cells. β2 microglobulin (B2M) is necessary to maintain the stability of HLA-I molecules, and mutations in B2M can lead to MHC instability and failure of antigen presentation. This mechanism has received more attention. In a study of NSCLC, acquired homozygous loss of B2M led to a lack of HLA-I molecule expression in tumors, and mouse models also exhibited resistance after the knockdown of B2M, suggesting that disruption of HLA-I molecule-associated antigen presentation can lead to ICIs resistance (42). The lack of HLA-I expression in small-cell lung cancer shows minimal ICIs activity, and HLA-I upregulation through IL-27/STAT3 activation can enhance ICIs efficacy (43). In addition, some studies suggest that 40% of NSCLC patients have a heterozygous loss of HLA, which is associated with tumor immune escape, but it is not clear how it relates to ICIs drug resistance (44).

The genotype of HLA may also influence the tumor response to ICIs. The anti-tumor activity of ICIs depends on CD8^+^ T cells and HLA-I-dependent immune activity (45). The HLA-I molecule is highly polymorphic, and each variant binds a particular peptide ligand. Thus, if HLA-I locus is heterozygous, it is most likely to bind the largest combinations of peptide ligands. In contrast, more presence of homozygous genes at the HLA-I locus theoretically is expected to provide relatively fewer combinations of MHC complex to CTLs (46). Studies have shown that maximal heterozygosity of HLA-I A/B/C improves overall survival (OS) after ICIs therapy, compared to patients with at least one homozygous HLA locus. Among NSCLC patients treated with nivolumab, those not expressing HLA-A*02 and alleles had inferior outcomes with progression-free survival (PFS) of 7.5 months, while HLA-A*-01 positive patients had the best outcome with a PFS of 22.6 months. Regarding the impact of heterozygosity on prognosis, heterozygotes in HLA-A were associated with worse OS, while heterozygotes in DRB1 prolonged OS, which highlights the influence of host germline genetics (47). Moreover, tumor cells may also express non-classical MHC-I antigens (like HLA-G, HLA-E), which can bind to inhibitory receptors on T cells and other immune cells to suppress cell function, causing ICIs resistance (23).

### 3.3 Inhibitory co-stimulatory factor expression

The expression of co-suppressor molecules in TME is the underlying mechanism of tumor immune escape, and targeting the PD-1/PD-L1 co-inhibitory axis demonstrates that blocking co-inhibitory signaling can reactivate T cells to mediate tumor elimination or control. However, the expression of co-inhibitory molecules other than PD-L1 on cancer cells could potentially lead to the failure of ICIs therapy.

Following the first series of inhibitory receptors (PD-1, PD-L1, CTLA-4), three new inhibitory receptors stood out: lymphocyte activation gene 3 (LAG-3), T cell immunoglobulin and mucin domain 3 (TIM-3), and T cell immunoreceptor with Ig and ITIM domains (TIGIT). LAG-3 is expressed on activated NK cells, Teffs, regulatory T cells (Tregs), B cells, and plasma cell-like DCs, preventing immune overactivation (48–50), TIM-3 has been identified on Tregs, DCs, NK cells, and monocytes, and has similar effects to LAG-3. TIGIT is also a co-inhibitory receptor on various immune cells (49). These molecules are promising therapeutic targets, and there is some evidence that they are associated with anti-PD-1 resistance. In 90 patients with advanced NSCLC treated with anti-PD-1 therapy, elevated LAG-3 expression was associated with PD-1 blockade insensitivity and resulted in shorter PFS (51). And in histology, LAG-3 enrichment suggests an immune contexture represented by CD8^+^ T cell dysfunction, which is associated with a worse prognosis (52). Another study also suggested anti-PD-1 resistant NSCLC harbored higher expression levels of TIM-3, LAG-3, and CTLA-4 on CD4^+^ and CD8^+^ T cells in, but only TIM-3 was significantly increased, suggesting the TIM-3 upregulation surrogate mechanism (53). It is noteworthy that studies on renal cell carcinoma (RCC) suggest that the expression of LAG-3, TIM-3, and TIGIT is exclusive at the single-cell level, with most suppressive costimulatory receptor-positive cells expressing mainly one of them, and no circumstance of three molecules are all low expressed. Among the three members, RCC with high LAG-3 expression had the worst survival rates and the most severe immunosuppressive microenvironment. It was resistant to anti-angiogenic therapy and ICIs, suggesting that tumor classification and outcome prediction can be based on dominant immunosuppressive molecules (54). TIGIT can also be elevated in NSCLC after resistance to anti-PD-1 therapies, but this remained controversial (42). Some clinical trials combining anti-PD-1 with anti-LAG-3/TIM-3/TIGIT have shown improved anti-PD-1 efficacy, providing strategies to overcome drug resistance (48, 55–57).

V-domain immunoglobulin suppressor of T cell activation (VISTA), or PD-1 homolog (PD-1H), is a novel inhibitory immune checkpoint, which is expressed on myeloid cells, lymphoid cells, and tumoral cells (58, 59). A study showed that VISTA was detected in 99% of NSCLC cases and predominantly expressed on stromal cells. But its expression was correlated spatially with immune infiltration and PD-1/PD-L1 expression, and regulated by local cytokines and IFN, suggesting that VISTA may not induce PD-1 resistance (60). However, upregulation of VISTA on infiltrating immune cells has been reported in prostate cancer treated with ipilimumab (anti-CTLA-4), suggesting its role in anti-CTLA-4 resistance (61). In addition, HMBD-002, an anti-VISTA antibody, can reduce myeloid-derived suppression of T cell activity and prevent neutrophil migration, stimulating a pro-inflammatory phenotype characterized by a Th1/Th17 response, making it a promising therapeutic target for ICIs combination (62).

CD91, also known as low-density lipoprotein receptor-related protein-1 (LRP-1), was a receptor expressed on antigen-presenting cells (APCs). Recent studies explored its role in immunosurveillance and found it was critical to trigger immune responses. Sedlacek AL et demonstrated that CD91 could not only activate NK cells through APCs *in vivo*, but also cytokines in NK cells. This procedure was essential for T cell and APC function (63). In addition, by stimulating the secretion of cytokines from macrophages, CD91 induced the activation of DCs to produce co-stimulation. When the expression of CD91 on DCs was depleted, mice were more likely to suffer the development of tumors induced by chemical compound. And in clinical practice, patients with advanced melanoma harboring high CD91 expression had better prognosis. Based on this, authors highlighted the potential function of CD91 in immunotherapy (64). However, it still needs further investigation. For example, heat shock protein (HSP) gp96 could combine with CD91. But the dose of gp96 had opposite roles, as low dose contributed to Th1 immune responses, and high dose induced immunosuppression *via* Tregs (65–67). Despite all this, the importance of CD19-mediated immune responses possess a variety of research potential.

### 3.4 Signaling pathway abnormalities

Lymphocyte infiltration and PD-L1 expression are vital factors for effective ICIs, and most tumors with anti-tumor immunodeficiency was lack of CD8^+^ T cell infiltration and tend to be resistant (68). Several signaling pathway abnormalities are associated with lymphocyte infiltration in TME and PD-L1 expression on the surface of cancer cells.

Existing evidence indicated that Wnt/β-catenin pathway activation can lead to non-inflammatory TME and cause anti-PD-1/PD-L1 resistance. In detail, β-catenin activation can lead to failure of DCs and effector T cell recruitment, resulting in impaired immune response (69–71). In addition, β-catenin can regulate Treg cell infiltration (72). PTEN is a negative regulator of the PI3K/AKT pathway, and PTEN loss leading to anti-PD1 resistance has been reported in various cancers, such as lung cancer and melanoma (70, 73). PTEN loss in tumor cells increases the expression of immunosuppressive cytokines, leading to reduced T cell infiltration in tumors and inhibition of autophagy. This could further reduce T-cell-mediated cytotoxicity and cause ICIs resistance (73–75). Moreover, patients carrying PTEN mutations could not benefit from ICIs, even if they possess high tumor mutation burden (TMB) and positive PD-L1 expression. But combined with temsirolimus (mTOR inhibitor), synergic therapeutic effects can be reached, which provided a combination option (76).

The RAS/MAPK pathway is a significant factor driving PD-L1 expression (77, 78), but its activation in triple-negative breast cancer is associated with reduced TIL infiltration (79, 80), which may cause immune escape and ICIs resistance. If MAPK activation causes low TIL infiltration and high PD-L1 expression simultaneously, it is ambiguous whether ICIs resistance could occur. In contrast, studies in melanoma suggest that MAPK pathway inhibition with targeting agents can lead to cross-resistance between targeted therapies and ICIs, following reduced infiltration of CD103 DCs (81), suggesting multiple functions of MAPK activation. In conclusion, the impact of MAPK on anti-PD-1 effects in NSCLC needs to be further investigated.

The PI3K/AKT/mTOR pathway was also associated with the expression of PD-L1. For example, by activating β-catenin and the downstream mTOR signaling, AKT pathway increased PD-L1 expression (82). Meanwhile, Liu M et al. found that PD-1 on myeloid-driven suppressor cells (MDSCs) could bind to PD-L1, and trigger PI3K/AKT pathway in B cells, which further impaired the function of T cells and led to tumor immune escape (83). It has been confirmed in a study that the combination of mTOR inhibitor (rapamycin) and anti-PD-1 blocked the progression of NSCLC (82). Currently, a phase I trial is active, which aims to evaluate the efficacy and safety of mTOR inhibitor combined with durvalumab for stage I-IIIA NSCLC (NCT04348292). We are looking forward to the results.

### 3.5 Interferon pathway

IFNs are essential for anti-tumor immune response and critical cytokines in cancer elimination. IFNs are classified into three types: type I (IFN-α, IFN-β, IFN-ϵ, IFN-κ, and IFN-ω), type II (IFN-γ), and type III (IFN-λ), which have multiple roles in anti-tumor cell proliferation, promotion of apoptosis, anti-angiogenesis, increasing antigen processing and presentation of APCs. Through multiple pathways, IFN can ultimately exert anti-tumor immune effects by increasing intrinsic and adaptive immune cell functions, and IFN mainly induces intracellular changes through the JAK/STAT pathway (84). Despite these positive effects of IFN, chronic IFN exposure exerts solid selective pressure on tumors and promotes tumor immune escape. In this process, tumors can not only upregulate the expression of immunosuppressive receptors and metabolites (PD-L1, CYLA-4, indoleamine 2, 3-dioxygenase (IDO)). but also downregulate JAK inhibitors, leading to sustained activation of the JAK/STAT3 pathway and promoting tumor growth (85). Sustained IFN exposure also attracts Tregs and suppressive MDSCs infiltration, leading to immunosuppression. Therefore, it is reasonable to discuss the effect of IFN on ICIs resistance.

The contribution of IFN to anti-PD-1 resistance is currently evaluated. On the one hand, the inactivation of the IFN effector pathway could impair IFN effects. For example, loss-of-function mutations in JAK1/2 can lead to a lack of PD-L1 expression, resulting in no response to ICIs, regardless of high TMB (86, 87). In addition, studies suggest that ICI-resistant melanomas contain B2M loss, which mediates resistance to CD8^+^ TIL, and JAK mutations create a severe pan-T cell immune escape phenotype (88). The second aspect is that IFN can initially induce anti-tumor effects but develop a range of mechanisms leading to resistance during ICIs treatment. Sustained IFN-γ signaling can lead to STAT1-related epigenomic changes to enhance the expression of IFN-stimulated genes (ISG), which leads to PD-L1 non-dependent resistance and multiple T cell inhibitory receptor ligands (89). Notably, another study suggests that STAT pathway silencing can lead to MHC II inhibition and immune escape, which may also be associated with ICIs resistance (88). Further exploration is necessary. In a mouse lung cancer model, IFN-γ was observed to cause phase separation of Yes-associated protein (YAP) to form condensation and translocate to the nucleus to form a potent transcription center, promoting the expression of multiple genes, including CD155 (ligand for TIGIT), leading to CTLs function inhibition and anti-PD-1 resistance (90). In addition, chronic IFN-I has been shown to trigger lipid peroxidation of terminal CD8^+^ T cells and accelerate their depletion, suggesting an effect of IFN on immune cell metabolism, which may also contribute to ICIs resistance (91).

### 3.6 Tumor microenvironment (TME)

TME is a highly heterogeneous environment composed of cancer cells, stromal tissue, extracellular matrix, and immune cells, characterized by high vascularization, glucose depletion, and hypoxia, where collective molecular signaling of immune cells affects tumor outcome by influencing the balance of inhibitory signaling and cytotoxic responses in the vicinity of tumor cells (23, 92). The acquisition and maintenance of cancer features, such as proliferative signaling, resistance to cell death, induction of angiogenesis, activation of invasion and metastasis, triggering tumor-promoting inflammation, and avoidance of immune destruction, rely to varying degrees on TME (93, 94). The immune system is an essential determinant of TME, and complex interactions between tumor cells and host immune responses are related to multiple components, including tumor parenchymal cells, fibroblasts, mesenchymal cells, blood, lymphatic vessels, TIL, chemokines, and cytokines.

Various components in TME can shape the immunosuppressive microenvironment and lead to ICIs resistance (23). Tregs negatively regulates the function of T cell (95). In cancer, Tregs mediate the dysfunction of the CD8^+^ T cells, which is characterized by upregulation of surface inhibitory receptors (e.g., CTLA-4). Furthermore, Treg-mediated IL-2 deprivation can exacerbate this dysregulation, leading to immune escape (96). Whether Tregs can cause ICI resistance is inconclusive, but some studies suggest its possible role in ICIs resistance. Co-blockade of PD-1 and PD-L1 in triple-negative breast cancer can upregulate the expression of immune checkpoints (e.g., CTLA-4, TIM-3, LAG-3) on Tregs, which could potentially lead to more severe T cell dysfunction through other co-inhibitory molecules expression (97). Moreover, selective inhibition of TGF-β1 released from Treg cells can overcome anti-PD-1 resistance (98), suggesting that TGF-β may be one of the reasons for Treg-mediated resistance (99). In addition, oxidative stress in TME can lead to Treg cell apoptosis. Apoptotic Tregs can release large amounts of ATP that further convert to adenosine *via* CD39 and CD73, exacerbating immunosuppression and eliminating PD-L1 blockade-mediated T cell activation (100).

TAMs are abundantly distributed and highly plastic in TME, and they are often defined as M1 and M2 subpopulations (101). M1 TAMs are the primary to trigger inflammation in the early stage of cancer. After that, TAMs undergo M2 polarization in response to signals in the TME (102). In contrast to tumor cells, PD-L1 expression on TAMs is mainly independent on local IFN-γ level. TAMs residing in TME have been shown to inhibit the anti-tumor effects of ICIs through multiple mechanisms (103), and TAMs enrichment-mediated anti-PD-1 resistance is independent of PD-L1 status (104). First, M2 TAMs can lead to T cell exclusion. In lung adenocarcinoma (LUAD), CD8^+^ T cell can interact with TAMs, leading to its poor migration and low CTLs infiltration in the TME. TAMs depletion reverses the circumstance and restore ICIs resistance (105). Additionally, induction of TAMs reprogramming to the M1 type could restore ICIs resistance, indirectly proving the role of M2 TAMs in ICIs resistance. Meanwhile, studies suggest that the re-polarization of M2 to M1 may cause PD-L1 upregulation, so ICIs combined with TAMs reprogramming may be a novel therapeutic strategy (106). Secondly, IDO is an essential immunomodulatory enzyme that inhibits T cell proliferation, promotes Treg cell differentiation, and induces TAMs and DCs to differentiate to suppressive phenotypes, producing potent immunosuppression (107). TAMs could in turn induce M2 transformation, exacerbating the immunosuppressive phenotype (89). Although various studies have demonstrated the contribution of IDO to T cell dysfunction, how M2 TAM-derived IDO-induced T cell tolerance contributes to PD-1/PD-L1 blockade resistance still needs to be thoroughly investigated.

MDSCs are heterogeneous bone marrow cells recruited to immunosuppressive TME. Some studies suggest that MDSCs may serve as a prognostic marker for ICIs, but the relationship between MDSCs and ICIs resistance has not been fully revealed. In NSCLC patients, the peripheral blood of non-responders had a higher frequency of MDSCs and fewer NK cells, compared with responders at the end of one round of PD-1 treatment (108). And high MDSCs in the peripheral blood was negatively associated with anti-PD-1 efficacy and prognosis (109, 110). Some studies also suggest that inhibition of MDSCs migration to TME may enhance the efficacy of anti-PD-1 and overcome PD-1 resistance (111–115). This evidence suggests that MDSCs may be involved in anti-PD-1 resistance. Theoretically, MDSCs, as a class of suppressive immune cell population, can interact with other immune cell components, secrete negative immune regulatory molecules, and are associated with resistance to chemoresistance and targeted therapy. Their relevant mechanisms in immunotherapy resistance deserve to be investigated (116–118).

### 3.7 Genetic mutations

It has been shown that genomic alterations in cancer cells can lead to ICIs resistance. Homozygous deletion of the 9p21.3 gene is one of the common genomic defects, accounting for approximately 13% of all cancers. 9p21 deletion is associated with a ‘cold’ tumor immune phenotype. This phenotype is characterized as reduced abundance of TIL (especially T/B/NK cells) immune cell trafficking and activation, PD-L1 positivity, and activation of immunosuppressive signaling. Clinical studies have demonstrated that patients with 9p21 loss have significantly lower response rates to ICIs and worse outcomes (119). In addition, KRAS mutations are common oncogenic mutations in NSCLC, and KRAS G12C mutations are associated with higher levels of inflammation in TME and better ICIs response (35, 120). However, conversely, KRAS-G12D mutations can suppress PD-L1 expression through the PI3K/AKT pathway and reduce chemokine CXCL10/CXCL11 levels through downregulation of high-mobility group AT-hook 2 (HMGA2), leading to reduced CTLs recruitment and ICIs resistance (121).

Serine/threonine kinase 11 (STK11) is a critical oncogene, and STK11 mutation is a common mutation secondary to KRAS mutation. Compared with wild-type STK11, NSCLC patients carrying STK11 mutations have a significantly lower OS rate (122). Genetic ablation of STK11 in animal models directly promotes resistance to anti-PD-1 therapies, and in a cohort of NSCLC patients, STK11^mut^ was associated with PD-L1 deletion, suggesting STK11 is related to PD-L1 expression and ICIs resistance. Moreover, STK11-deficient tumors were associated with a low density of CTLs. However, the negative impacts of STK11/LKB1 genomic alterations on resistance to ICIs are also seen in PD-L1 positive NSCLC, suggesting that PD-L1 expression is partially dependent on STK11 (123). In the periphery, STK11^mut^ tumors have lower NK cells and CD4^+^/CD8^+^ effector memory T cells. In TME, there is a significant upregulation of neutrophil-related markers (CXCL2, IL-6, CSF3) and inhibitory immune checkpoint killer Ig-like receptor (KIR), indicating the poor outcome of STK11 mutation may be attributed to enrichment of immunosuppressive mechanisms (124).

Moreover, kelch-like ECH-associated protein 1 (KEAP1) mutation can be seen in 20% of NSCLC and is the third most frequently mutated gene in LUAD, with a common substrate of neuropilin 2 (NRP2) (125). And KEAP1 is like to co-mutate with STK11 and KRAS (126). A study suggests that KEAP1 mutation in NSCLC is correlated with inferior ICIs response (127), and patients carrying both STK11 and KEAP1 mutations had worse outcomes compared with those harboring one mutation, suggesting an additive effect of these mutations (128, 129). Despite KEAP1 show its role in chemotherapy resistance (130), the contribution of KEAP1 mutation to ICIs resistance is unclear. The study shows that patients who harbored KEAP1 mutation had higher PD-L1 expression, compared with wild type, which may assist ICIs efficacy (131). But other studies also point out that the contribution of KEAP1 mutation to ‘cold’ microenvironment was characterized by failure in IFN expression, innate immune cell recruitment and TIL infiltration (132–136).

There are some ‘driver mutations’ in cancer that are responsible for both the initiation and maintenance of the malignancy. Receptor protein tyrosine kinases like EGFR and ALK are typical examples, whose mutations are related to ICIs resistance (137). The EGFR signaling pathway is one of the most important oncogenic pathways in NSCLC (138). Recent studies demonstrated their impact on the immune system was apart from tumor biology, and relationships between activation of EGFR and PD-L1 upregulation have been widely validated clinically in NSCLC patients (139, 140). But under high PD-L1 circumstances, EGFR-mutant cancer is insensitive to ICIs. Studies show that EGFR-mutant and ALK-positive NSCLCs lack concurrent PD-L1 expression and high levels of CD8^+^ TILs (141). EGFR mutation is correlated with uninflamed TME with immunological tolerance and weak immunogenicity, resulting in an inferior response to PD-1 blockade (142).

Tumors carrying oncogenic mutations usually follow the principle of oncogenic addiction, which means that the evolution of these tumors depends on the specific activity of an activated oncogene that confers a survival advantage to tumor cells (143, 144). Consequently, these tumors were usually characterized as low TMB and ‘cold’ TME, which is related to ICIs resistance (144, 145). For driver mutation-positive NSCLC, target therapy is currently the optimal choice. Considering the effect of driver mutations on the immune system, combining targeted therapy and immunotherapy may be feasible. Unfortunately, the therapeutic role of ICIs in oncogene-driven NSCLC remains unclear. The level of evidence supporting the use of immunotherapy for patients with NSCLC harboring driver mutations is quite low, which is because patients with EGFR or ALK alterations are often excluded in most ICIs trails (144). Some clinical trials that expect to find rationale to combine tyrosine kinase inhibitor (TKI) and ICIs show limited clinical benefits but with more adverse events (146, 147) (**Table 1**). Therefore, more factors need to be considered to determine the feasibility of combining ICIs and targeted therapy.

**Table 1:** Clinical trials investigating the effects of ICIs in driver mutation-positive NSCLC.

Study	Patients	Strategy	Results	Reference
TATTON (NCT02143466)	EGFR^+^	Osimertinib plus Durvalumab Group A: progress on a previous EGFR TKI Group B: first-line osimertinib plus durvalumab	Group A: ORR:43%, mDOR: 20.4m; Group B: ORR: 82%, mDOR: 7.1m	146
CheckMate-012 (NCT01454102)	EGFR^+^	Nivolumab + Erlotinib	ORR: 15%; 24-week PFS rate: 48%	148
-(Safety evaluation)	ALK^+^/ROS^+^/MET^+^	ICI + Crizotinb	ICI + Crizotinb: increase ALT level: 45% increase AST level: 36.4% Crizotinib alone: increase ALT level: 8.1%. increase AST level: 3.4%	149
COSMIC-021 (NCT03170960)	EGFR^-^/ALK^-^/ROS1^-^	Cabozantinib + Atezolizumab (C+A) *Vs* Cabozantinib (C)	C+A: ORR: 19%; DCR: 80%; mOS: 13.8m C: ORR: 6%; DCR: 65%; mOS: 9.4m	150
WJOG8515L	EGFR^+^ (EGFR TKI resistant)	Nivolumab (N) *vs* Pemetrexed + Carboplatin (PC)	N: mPFS: 1.7m; OS: 20.7m; P+C: mPFS: 5.6m; OS: 19.9m	151
CheckMate-722 (NCT02864251)	EGFR^+^ (EGFR TKI resistant)	Nivolumab + Chemotherapy	Active	–
KEYNOTE-789 (NCT03515837)	EGFR^+^ (EGFR TKI resistant)	Pemetrexed + Platinum *vs* Pemetrexed + Platinum + Pembrolizumab	Active	–

ICI, immune checkpoint inhibitor; TKI, tyrosine kinase inhibitor; ORR, objective response rate; DOR, duration of response; PFS, progression-free survival; ALT, alanine aminotransferase; AST, aspartate aminotransferase; OS, overall survival

### 3.8 Metabolism

Metabolic reprogramming is considered a new hallmark in cancer (152). Metabolic reprogramming was essential for the proliferation, differentiation, and various functional assays of immune cells (153, 154). In addition, nutrient depletion, hypoxia, reactive nitrogen, and oxygen intermediates production in TME force immune cells to undergo metabolism reprogramming, which further impair immune cell function and cultivate an immunosuppressive TME (155–157). Metabolites in the TME can, in turn, affect immune cell differentiation and their function (158). It has been shown that PD-1 can lead to an immunosuppressive landscape through immune cell metabolism reprogramming, and ICIs application can reinvigorate the immune response (159, 160). Based on this, it is rationale to discuss the relation between ICIs resistance and cancer metabolism.

Existing evidence suggested that oxidative metabolism and hypoxia may contributed to ICIs resistance. In head and neck squamous carcinoma, tumors resistant to ICIs showed a more active oxidative metabolism, leading to TME hypoxia and a decrease of CD8^+^ T cells (161). Studies in melanoma also shown that enhanced tumor oxidative metabolic activity contributed to increased oxygen consumption, which correlated with T cell depletion and reduced immune activity (162, 163). In conclusion, tumors resistant to ICIs was characterized as a ‘hypermetabolic’ phenotype, with an upregulation of oxidative phosphorylation activity. On the one hand, such phenotype exacerbated hypoxia, which contributed to T cell apoptosis by inhibition of CCR7 and NK cells (164). Meanwhile, tumor cell hypermetabolism may lead to T cell metabolic starvation, resulting in loss of glycolytic potential (163). Although how PD-1 treatment impacts tumor oxidative metabolism is unknown, these studies suggest that TME hypoxic state after ICIs treatment could be served as a potential prognostic biomarker.

T cell metabolism may relate to ICIs resistance. It was established that PD-1 signaling activation can attenuate T cell glycolysis, glutaminolysis, and branched-chain amino acid metabolism. It can also affect mitochondrial sub-microstructure, leading to reduced mitochondrial depolarization and mitochondrial dysfunction, which could impair cellular oxidative phosphorylation. In contrast, fatty acid oxidation was enhanced during the management of ICIs (165, 166). Overall, PD-1 signaling activation altered T cell metabolic activity, which prevents terminal differentiation and glycolysis-induced death of T cells (166). Consequently, blockading PD-1 signaling with ICIs may accelerate apoptosis and deletion of Teffs, resulting in no response to ICIs.

Glutamine metabolism is essential for tumors, while glutamate is necessary for T cell activation and expansion (167). When tumor cells competitively intake glutamine could lead to T cell dysfunction and ICIs resistance (168). A subset of lung squamous carcinoma resistant to ICIs was defined as the hypermetabolic, which was dependent on glucose and glutamine. When glutamine intake was inhibited, the effect of ICIs was restored. These findings established a cornerstone that the inhibition of glutamine metabolism may enhance the efficacy of ICIs (169). In addition, after inhibiting the glutamate/cystine reverse transporter, melanoma cells secrete large amounts of PD-L1 in the form of exosomes to induce M2 polarization of TAM, leading to ICIs resistance (170). Combined with the evidence above, high glutamate in tumor cells is suggested to be detrimental to ICIs response.

Moreover, glutamate has novel atypical functions. Cancer cells with abnormal glutamate decarboxylase (GAD) expression could synthesize γ-aminobutyric acid (GABA). Subsequently, GABA binds and activates GABA^B^ receptors to inhibit GSK-3β and enhance β-catenin signaling, stimulating tumor cell proliferation, and inhibiting CD8^+^ T cell infiltration, leading to anti-PD-1 resistance (171).

Disturbances of lipid metabolism also contribute to the development of cancer, as well as ICIs resistance. First, it has been mentioned above that chronic IFN can lead to lipid peroxidation in terminal CD8^+^ T cells and accelerate their depletion (91). Second, oxidized low-density lipoprotein (LDL) in serum not only suppresses T cell function but also upregulates heme oxygenase 1 (HO-1) expression, which induces a cytoprotective stress response to avoid cancer cell apoptosis and leads to ICIs resistance (172). Furthermore, cholesterol is vital in T cell function and ICIs sensitivity. Elevated cholesterol in T cells upregulates the expression of various immunosuppressive receptors (e,g, PD-1, TIM-3, LAG-3) and impairs ferroptosis of tumor (173). It also modulates the endoplasmic reticulum stress pathway to inactivate T cells (174), leading to immune depletion. Moreover, inhibiting cholesterol esterification in T cells by acetyl-CoA acetyltransferase (ACAT1, a cholesterol esterase) enhances the effector function and proliferation of CD8^+^ T cells. It enhances ICIs effect due to elevated cholesterol levels in the CD8^+^ T cell plasma membrane, which enhances T cell receptor aggregation and synapse formation (175).

Although many studies suggest a role for metabolism in immunosuppression, and targeting metabolism can improve ICIs effect, they do not demonstrate that metabolic profiles can distinguish ICIs responders from non-responders (176). In addition, current studies are preclinical, it is unclear whether targeting metabolic pathways will promote ICIs response in clinical practice. More definitive mechanisms of resistance remain to be explored.

### 3.9 Microbiota

Microbiota, has a vital role in various aspects of human physiology. The gut microbiota has a significant role in regulating host metabolism and directing the development and function of immune system. Studies suggest that the primary response to ICIs in patients with epithelial tumors and melanoma may benefit from the gut microbial ecosystem (177–180). Several clinical and preclinical evidence has validated the gut microbiota’s impact on ICIs efficacy (178–183).

Microbiota composition is related to ICIs sensitivity. Combining anti-PD-1 with fecal microbiota transplantation (FMT) from healthy donors could enhance sensitivity to anti-PD-1 treatment (184). The antibiotic application which could change microbiota composition may impair the efficacy of ipilimumab. Moreover, anti-CTLA-4-induced TIL infiltration was significantly reduced in germ-free mice (180). Clinical trials of FMT in combination with anti-PD-1/PD-L1 also showed promising results, as 6 of 15 patients benefitted from it. Increased CD8^+^ T cell activation and reduced myeloid cell were identified (185). These suggest that microbiota is necessary for ICIs treatment. Moreover, CTLA-4 blockade reverses the gut microbiota repertoire, leading to an increase of bacteria, which diminishes the effects of ipilimumab. And this provided new insights into acquired resistance to anti-CTLA-4 (186).

The efficacy of ICIs in epithelial tumors was correlated with the abundance of Akkermansia muciniphila (AKK) (179), and the study in advanced NSCLC conquered the conclusion (177). AKK-free feces can confer resistance to anti-PD-1 in mice, and the baseline expression of AKK is positively correlated with increased ORR and OS. on the other hand, studies suggest that inosine, a metabolite of Bifidobacterium pseudolongum, can interact with T cell A^2A^ receptors to enhance anti-CTLA-4 effects (187). In advanced NSCLC, patients with abundant Bifidobacterium breve have superior PFS than patients with undetectable bifidobacteria (188).

In colon cancer, intestinal innate lymphoid cell 3 (ILC-3) was severely dysregulated. ILC-3 can interact with T cells *via* MHC-II to induce microbiota colonization, eliciting type I immune responses. Transferring microbiota from patients with dysregulated intestinal ILC-3 to mice induces ICIs resistance, suggesting that immune cells can directly influence intestinal microbiota colonization, which in turn affects the anti-tumor immune response (189).

In all, current evidence shows that bacterial species and microbiota composition are associated with ICIs effect. Moreover, an increasing number of studies suggest that microorganisms may be present in tumor tissues, and involved in tumor progression and ICIs resistance (190, 191). The mechanisms remain unclear. However, some mechanisms have been proposed, such as cross-reaction between cancer antigens and microbial peptides, direct presentation of intracellular bacterial peptides by cancer cells (25, 187, 189, 192). Based on this, relative therapeutic approaches have been proposed, such as fecal microbiota transplantation, bacterial isolates and consortia, probiotic therapy (190). More research is still needed to determine microbiota’s role in immunotherapy and ICIs resistance.

### 3.10 Other potential mechanisms

Epigenetics has a vital role in regulating gene expression (193). Anti-Silencing Function 1A Histone Chaperone (ASF1A) encodes a member of the H3/H4 family of histone chaperone proteins and is a critical component of the histone donor complex during nucleosome assembly. It also binds to bivalent promoters and transcription factors to regulate gene expression (194, 195). ASF1A deficiency sensitizes LUAD to anti-PD-1 by promoting M1 TAM polarization and T cell activation, suggesting that ASF1A may be a regulator of anti-PD-1 therapeutic sensitivity (194).

In addition, it has been shown that a secreted PD-L1 splicing variant, which lacks a transmembrane structural domain, competitively binds anti-PD-L1 monoclonal antibodies, neutralizing their effects and leading to drug resistance in NSCLC (196).

## 4 Firing up the cold tumor: Strategies overview

A classification has been proposed based on the clinical response to ICIs treatment. Tumors are considered ‘hot’ if they respond to ICIs and ‘cold’ was characterized as the lack of HLA-I expression, low PD-L1 expression, and infiltration of immunomodulatory cells (197). The third category of ‘warm’ tumors has also been proposed, but this group lacks sufficient clinical information details (12, 198). However, this classification remains controversial. For example, tumor can have a low initial antigen presentation effect by reducing neoantigen expression, resulting in reduced T cell activation, or tumor overexpressing multiple suppressive immune checkpoint molecules. Both of them were identified as ‘cold’ tumors. but the response to ICIs cannot be generalized. Therefore, how to fire up ‘cold’ tumors is the key to overcoming ICIs resistance.

Combination therapy is the most common treatment, but it should be based on an in-depth understanding of the mechanisms of ICIs resistance, rather than simply combining existing therapies (25). Radiotherapy/chemotherapy combined with anti-PD-1/PD-L1 therapy has become the standard treatment for advanced NSCLC. Its rationale has been elaborated, such as enhancing tumor antigenicity, modulating suppressive immune cells, promoting pro-inflammatory factor release and antigen presentation (11, 199, 200). A large number of clinical trials have demonstrated its effectiveness (201, 202). In addition, some studies suggest that ICIs therapy is ineffective after first-line chemotherapy, and transcriptional analysis shows significant downregulation of gene expression related to antigen processing and presentation, as well as IFN and chemokine-related pathways, suggesting that immunotherapy after chemotherapy is not beneficial (203). In addition, anti-PD-1/PD-L1 combined with anti-CTLA-4 has complementary anti-tumor mechanisms. Ipilimumab combined with nivolumab has been approved for the treatment of advanced melanoma, renal cell carcinoma, and metastatic colon cancer (204). Clinical trials in NSCLC have also shown promising results. Checkmate-227 showed superior outcomes of nivolumab in combination with ipilimumab, compared to chemotherapy, especially for patients with TPS ≥ 50% (205). Another phase II clinical trial investigated the impact of durvalumab in combination with tremelimumab in NSCLC patients resistant to anti-PD-1, with 10% overall response rate of and 30% disease control rate (206).

In addition, there are many novel drugs for combination therapy, such as targeted therapies (207–210), lysing viruses (211), cancer vaccines (212), Chinese herbal medicine (213, 214), and nanoparticles (215, 216). Meanwhile, metabolism and microbiome with immunotherapy is ongoing. For example, inhibition of mitochondrial metabolism using atovaquone reduces MDSCs and Tregs infiltration, while CD4^+^ TIL infiltration increases (217). Targeting the fatty acid metabolizing enzyme Stearoyl-CoA desaturase-1 (SCD1) combined with anti-PD-1 also have a synergistic anti-tumor effect, by reducing Wnt/β-catenin signaling and promoting DCs recruitment (218). Although some studies showed promising results, there are still many challenges, such as the double-edged impact of glutaminase antagonism on T cell (167, 219, 220). The combination of IDO inhibitors with ICIs did not provide clinical benefit for patients (221). Moreover, several combination therapies are partly achieved by intratumor injection, limiting its clinical application. Therefore, more exploration is necessary.

## 5 Summary and perspective

Although ICIs have led to a paradigm shift in cancer treatment, resistance to ICIs has limited its application and the mechanism represents a critical knowledge gap. Here, we discussed ICIs resistance mechanisms. Briefly, factors such as neoantigen presentation, epigenetic modifications, tumor heterogeneity, quantity, and quality of immune cells in TME simultaneously determine the response to ICIs therapy (2). However, the interaction of these factors increases the complexity of the study, and there are still many mechanisms need to be explored.

Although significant efforts have been made to overcome the resistance to ICIs, but up to the present they remain investigational and need to be confirmed in the future.

## Author contributions

KZ, SL and KC contributed to conception and design of the study. KZ wrote the first draft of the manuscript. SL and YZ wrote sections of the manuscript. KC reviewed the manuscript. All authors contributed to manuscript revision, read, and approved the submitted version.
